# Environmentally azithromycin pharmaceutical wastewater management and synergetic biocompatible approaches of loaded azithromycin@hematite nanoparticles

**DOI:** 10.1038/s41598-022-14997-y

**Published:** 2022-06-29

**Authors:** Mostafa F. Al-Hakkani, Gamal A. Gouda, Sedky H. A. Hassan, Mahmoud M. A. Mohamed, Adham M. Nagiub

**Affiliations:** 1 Department of Chemistry, Faculty of Science, Al-Azhar University, Assiut Branch, Assiut, 71524 Egypt; 2 Department of Chemistry, Faculty of Science, New Valley University, El-Kharja, 72511 Egypt; 3 Department of Botany and Microbiology, Faculty of Science, New Valley University, El-Kharja, 72511 Egypt; 4 Department of Biology, College of Science, Sultan Qaboos University, Muscat 123, Oman

**Keywords:** Chemical biology, Chemistry, Materials science, Nanoscience and technology

## Abstract

Pharmaceutical wastewater contamination via azithromycin antibiotic and the continuous emergence of some strains of bacteria, cancer, and the Covid-19 virus. Azithromycin wastewater treatment using the biosynthesized Hematite nanoparticles (α-HNPs) and the biocompatible activities of the resulted nanosystem were reported. Biofabrication of α-HNPs using *Echinacea* *purpurea* liquid extract as a previously reported approach was implemented. An evaluation of the adsorption technique via the biofabricated α-HNPs for the removal of the Azr drug contaminant from the pharmaceutical wastewater was conducted. Adsorption isotherm, kinetics, and thermodynamic parameters of the Azr on the α-HNPs surface have been investigated as a batch mode of equilibrium experiments. Antibacterial, anticancer, and antiviral activities were conducted as Azr@α-HNPs*.* The optimum conditions for the adsorption study were conducted as solution pH = 10, 150 mg dose of α-HNPs, and Azr concentration 400 mg/L at 293 K. The most fitted isothermal model was described according to the Langmuir model at adsorption capacity 114.05 mg/g in a pseudo-second-order kinetic mechanistic at R^2^ 0.9999. Thermodynamic study manifested that the adsorption behavior is a spontaneous endothermic chemisorption process. Subsequently, studying the biocompatible applications of the Azr@α-HNPs*.* Azr*@*α-HNPs antibacterial activity revealed a synergistic effect in the case of Gram-positive more than Gram-negative bacteria. IC_50_ of Azr*@*α-HNPs cytotoxicity against MCF7, HepG2, and HCT116 cell lines was investigated and it was found to be 78.1, 81.7, and 93.4 µg/mL respectively. As the first investigation of the antiviral use of Azr@α-HNPs against SARS-CoV-2, it was achieved a safety therapeutic index equal to 25.4 revealing a promising antiviral activity. An admirable impact of the use of the biosynthesized α-HNPs and its removal nanosystem product Azr@α-HNPs was manifested and it may be used soon as a platform of the drug delivery nanosystem for the biomedical applications.

## Introduction

Recently, the world is witnessing great development and a faster race in the fields of pharmaceutical industries. This is evident in the circumstances of the emerging coronavirus and different cancer diseases. These difficult circumstances demonstrated the strength of the solidarity and complementarity of the various sciences in scientific research to find a safe way out in light of this global crisis. Several studies have been shown investigating a solution to this pandemic the dual role and the great efficacy of many antibiotic drugs^1–7^. Recent studies have shown the effectiveness of some antibiotics from the macrolide family, such as azithromycin (Azr)^3,6,8^, and the cephalosporin family, such as ceftriaxone and cefixime (Cfx)^9–12^, for use as an antidote to some cancerous and viral diseases. From this standpoint, we would like to draw attention to the fact that this effect could be multiplied when it is integrated and installed or biocompatible via nano-drug-delivery to make the most of the involvement of the carriers at the nanoscale^7,13^. Also, nanomaterials have received wide attention in recent studies in entering the racetrack as an alternative, fast, effective, and safe drug to overcome these new diseases that harm the survival of the human race. This associated significance according to their unique physicochemical as particle size, shape, surface area, and surface potential^7,14^.

The world has witnessed the widespread use of macrolide and cephalosporin antibiotic medicines; consequently, large quantities of the production are required for these drug products to meet urgent needs. This large-scale use has contributed to increasing pollution of the aquatic environment from the wastewater of the pharmaceutical factories and hospitals. The high excessive consumption of these antibiotics may lead to necrosis of the renal tubules^7,15^.

**Figure 1:**
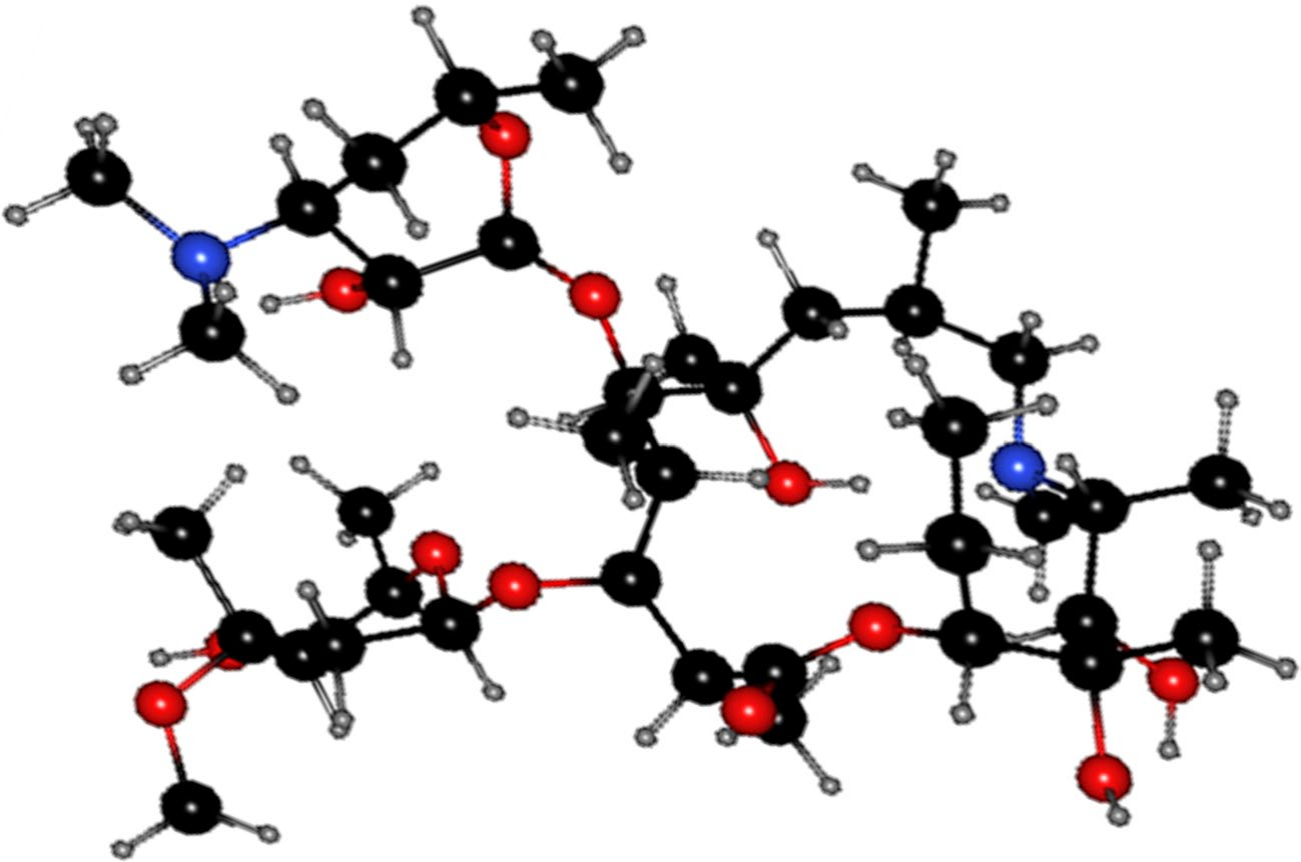
Structure of Azithromycin (C_38_H_72_N_2_O_12_, molecular mass 748.996 g/mol).

Azr is a member of the macrolide antibiotics, semi-synthetic, and is derived from erythromycin Fig. 1. Azr is extremely lipophilic with limited antibiotic water solubility and a fairly weak oral bioavailability of 37% after ingestion^16,17^.

Azr is used to treat multiple and diverse diseases of the bacteria, such as Gram-positive and Gram-negative microorganisms. Nitrogen atom involvement in the ring causes major improvements in the Azr pharmacokinetics, microbiological, and chemical properties. It is available in different oral dosage forms; powder for infusion solution, oral suspension powder, tablet, and capsules^16^.

Azr can be removed using several physicochemical methods or techniques such as the photocatalytic degradation process^7,18–20^, and advanced oxidation with the ozonation process^21,22^. Adsorption is easy to perform in these processes, it has cost-effective, high performance, and poses no risk of highly toxic by-products. It can also be considered one of the safest and most effective strategies for removing antibiotics from aqueous environments^23^. In the removal of Azr from aqueous solutions, various adsorbents have been widely used at the nanoscale^20,24–28^.

In the purification of water, metal oxide nanoparticles are mostly used as filters or may be mixed into a polymer matrix. Such represents ceramic materials enhance the isolation and the microstructure of the polymeric membrane^23,28–30^. The desirable characteristics of iron oxide nanoparticles such as high surface area, strong adsorption capacity to organic pollutants, and more specifically, the ability to magnetize; all these characteristics have recently given iron oxide nanoparticles much attention^7,31^.

The present approach assesses the removal and remediation of the Azr antibiotic via adsorption technology using nano-bioadsorbents. According to our literature survey, this research could be the first study that utilized the removal of the Azr contaminant from the aquatic environment using the biofabricated Hematite nanoparticles (α-HNPs).

The importance of the current research lies in the optimal use of α-HNPs in terms of their quadruple use; firstly, as a bio-adsorbent used to get rid of traces of the Azr antibiotic found in the wastewater of pharmaceutical factories. Secondly, the use of the Azr*@*α-HNPs as an antibacterial vehicle against Gram-positive and Gram-negative bacteria. Thirdly, the synergetic comparative efficacy of the combination of Azr*@*α-HNPs against the use of α-HNPs alone as anticancer. Finally, utilization of the combination of Azr*@*α-HNPs as nano-drug-delivery in studying its effectiveness in confrontation with the Coronavirus as an antiviral.

## Materials and methods

### Chemicals and reagents

All chemicals used in the current work were analytical grade. Azr standard (C_38_H_72_N_2_O_12_; molecular mass 748.996 g/mol; purity 98.4%; Century pharmaceuticals LTD., India), potassium dihydrogen phosphate, sodium hydroxide (Scharlau, Spain), hydrochloric acid; purity 37%, acetonitrile, and methanol HPLC grade from (Merck, UK). Phosphate buffer pH 8.0 was prepared by mixing 100 mL of 0.2 M potassium dihydrogen phosphate and 93.6 mL of sodium hydroxide 0.2 M, then diluted to 1000 mL with deionized water.

### Methods

#### Instrumentation

Most of the characterizations and analyses were conducted at the Central Laboratory for Microanalysis and Nanotechnology, Minia University, Egypt. The α-HNPs and Azr*@*α-HNPs were characterized via several instrumental analysis tools starting with the use of the digital balance of 5 digits [CX 265] (Citizen, India), and Eppendorf Centrifuge [5425] (Life science, Germany). FT-IR analysis was recorded on [Nicolet iS10 FT-IR spectrometer] (Thermo Fisher, USA) in a wavenumber range of 4000–400 cm^−1^ using an ATR module. The morphology of the α-HNPs and Azr*@*α-HNPs were investigated using scanning electron microscopy [SEM; JSM IT 200] (JEOL, Japan) and transmission electron microscopy [TEM; JEM-100C XII)] (JEOL, Japan), pH-meter [SevenMulti] (Mettler Toledo, Switzerland). Azr quantitative analysis was determined using the LC-20A HPLC instrument with the PDA (Shimadzu, Japan). The method was performed on the reversed-phase column ODS-3 (250 mm × 4.6 mm × 5 μm) (Thermo Scientific, USA).

#### Bioadsorbent preparation and characterization

The as-biofabricated α-HNPs were prepared and characterized using various instrumental techniques as in our previously reported green biosynthesis approaches^7,29^.

#### Batch adsorption mode experiments

As a general operating procedure, the para-film was used as a preventive action to avoid the adsorbate evaporation during the adsorption process. All tests were performed at least in triplicates with a relative standard deviation of less than 3 percent to assure the repeatability, robustness, and ruggedness of the implemented adsorption process according to the validation guidelines^32–36^. The test after adsorption was filtered through a nylon filter paper 0.45 µm pore size using the Buckner filtration system, then a further filtration was conducted using a syringe filter of 0.2 µm before introduction to the HPLC instrument. Initial and final concentrations of Azr liquid adsorbate were estimated using the previously validated HPLC method as reported by Al-Hakkani^35^.

##### Interactive effect of the change in solution pH

The adsorption experiment was conducted firstly to achieve the most convenient pH circumstances to get out the highest removal percentage of Azr according to the following equations^23^:

1 $$q_{e} = \, \left( {C_{0} - C_{e} } \right)xV \, / \, m.$$

q_e_ is adsorbed amount of Azr (mg/g); C_0_ is the initial concentration of Azr (mg/L); C_e_ represents the equilibrium concentration of Azr (mg/L); V is the volume of the Azr (L); m is the α-HNPs mass (g).

2 $$Adsorption\,\left( \% \right) \, = \, \left( {C_{0} - \, C_{e} } \right) \, /C_{0 } \times \, 100.$$

The pH study was conducted at different values in the range (2.0–10.0) of pH for Azr solutions maintaining other of the reaction conditions fixed at 293 K, 50 mL of Azr concentration 100 mg/L in Erlenmeyer flasks, 50 mg of α-HNPs through at 300 min with constant stirring at 500 rpm. The adjustment of solution pH was achieved using 0.1 M HCl or 0.1 M NaOH to reach the desired pH of the solution.

##### Adsorbent dose effect

The adsorption process was investigated to achieve the best result of the adsorbate removal at different adsorbent masses of the α-HNPs in the range of 50–200 mg with fixing the rest adsorption process parameters at 293 K, 50 mL of Azr concentration 100 mg/L at pH solution 10, and full time 300 min with constant stirring at 500 rpm.

##### Isothermal models study and adsorbate concentration effect

Different concentrations of 100 mL Azr (100–1000) mg/L at pH 10, 150 mg mass of α-HNPs as adsorbent through a full reaction time of 300 min with constant stirring at 500 rpm, and the temperature at 293 K was being investigated to determine the ideal convenient isothermal model of the adsorption process.

In trying to understand the relationship between adsorbate/adsorbent, four different isothermal models were studied using Langmuir, Freundlich, Temkin, and Dubinin–Radushkevich (D–R) isothermal models. Also, the best and most accurate, suitable model for assessing the adsorption process for Azr adsorption on the *α*-HNP surfaces was investigated as the following equations:

Langmuir model^37^:

3 $$C_{e} /q_{e} = \, \left( {1/q_{L} K_{L} } \right) \, + \, \left( {1/q_{L} } \right)C_{e} ,$$

4 $$R_{L} = \, 1/ \, \left( {1 + K_{L} C_{max} } \right).$$

q_L_ is the monolayer adsorption capacity of α-HNPs (mg/g); K_L_ represents Langmuir energy of adsorption constant (L/mg); R_L_ is the separation factor; C_max_ acts as the highest initial Azr concentration in the solution (mg/L).

Freundlich model^38^:

5 $$log \, q_{e} = \, log \, K_{F} + \, \left( {1/n} \right) \, log \, C_{e} .$$

K_F_ represents the Freundlich adsorption capacity of α-HNPs (mg/g); n is the Freundlich constant characteristics of the system, indicating the adsorption intensity.

Temkin model^39^:

6 $$q_{e} = \, B_{T} ln \, A_{T} + \, B_{T} ln \, C_{e} ,$$

7 $$b_{T} = \, RT/ \, B_{T} .$$

A_T_ is the binding constant (L/mg) and it was related to the maximum binding energy; B_T_ is Temkin adsorption constant (KJ/mol) that is related to the sorption heat; R represents the gas constant (8.314 J/mol K); T expresses the absolute temperature at 298 K; b_T_ is the adsorption process constant.

Dubinin–Radushkevich (D–R) model^28^:

8 $$ln \, q_{e} = \, ln \, q_{m} {-}\beta \varepsilon^{2} ,$$

9 $$\varepsilon \, = \, RT \, \left( {1 + \, 1/C_{e} } \right),$$

10 $$E_{D} = \, \left( { - 2 \, \beta } \right)^{ - 1/2} .$$

q_m_ is the D–R adsorption capacity of α-HNPs (mg/g); β represents the mean free energy parameter (mol^2^/KJ^2^); ε acts as the Polanyi potential; E_D_ parameter determines the adsorption energy per molecule of the Azr when it is transferred to the surface of the solid α-HNPs from infinity in the solution (kJ/mol).

##### Thermodynamic and kinetic studies

Temperature and contact time dependence effects for thermodynamic and kinetic studies also were to be evaluated. Studies were proceeded at different temperatures in the range 293–323 K and investigated according to thermodynamics equations as follows:

11 $$\Delta G \, = \, - \, RT \, ln \, K_{c} ,$$

12 $$ln \, K_{c} = - \, \Delta G/ \, RT \, = \, - \, \left( {\Delta H/ \, RT} \right) \, + \, \left( {\Delta S/R} \right),$$

13 $$K_{c} = \, C_{ads} / \, C_{e} .$$

∆G determines the free energy change (J/mol); R is gas constant (8.314 J/mol K); T represents the absolute temperature (K); K_c_ describes the thermodynamic equilibrium constant; ∆H determines the enthalpy change (J/mol); ∆S represents the entropy change (J/mol K); C_ads_ acts the concentration (mg/L) of the adsorbed Azr.

The time intervals range was studied between 2 and 300 min with reserving other of the reaction conditions constant and initial Azr concentration of 400 mg/L. Four different kinetic models were being applied for the investigation the analysis of the kinetic data to find the best convenient fitted model for describing the kinetic mechanism of the Azr adsorption onto the surfaces of the α-HNPs.

The adsorption efficiency %, isothermal, kinetics, and thermodynamics parameters of each study were being evaluated and investigated according to the equation lists as the following equations:

Pseudo-first-order model is by the Lagergren model^40^:

14 $$log\left( {q_{e} - q_{t} } \right) \, = \, log \, q_{e} {-} \, \left( {K_{1} /2.303} \right) \, t.$$

Pseudo-second-order by McKay and Ho model^41^:

15 $$\left( {t/q_{t} } \right) \, = \, 1/ \, \left( {K_{2} q_{e}^{2} } \right) \, + \, \left( {1/q_{e} } \right) \, t.$$

Elovich model^28^:

16 $$q_{t} = \, (1/\beta ) \, (ln\alpha \beta ) \, + \, (1/\beta ) \, ln \, \left( t \right).$$

Weber’s and Moris’s/intraparticle diffusion model^42^:

17 $$q_{t} = \, C \, + \, K_{int} \left( t \right)^{1/2} .$$

C_e_ is the equilibrium concentration (mg/L) of the remained Azr in solution after the adsorption process; q_e_ represents the amount of Azr contaminant adsorbed by α-HNPs (mg/g) at equilibrium; q_t_ represents the amount of Azr adsorbed by α-HNPs (mg/g) at predetermined time interval t; K_1_ describes the rate constant of pseudo-first-order adsorption process (min^−1^); t is the time interval (min); K_2_ describes the rate constant of pseudo-second-order adsorption process (g/mg min); *α* acts as the initial sorption rate constant (mg/g min); β acts as the constant related to surface coverage and the activation energy for chemisorption (g/mg); K_int_ describes the intraparticle rate constant (mg/g min^1/2^); higher values of K_int_ reflect an enhancement in the adsorption rate of Azr onto the α-HNPs surface; C is the value gives information about the boundary thickness, as the larger the intercept, the greater is the boundary layer effect.

#### Regeneration of the adsorbent

Regeneration of the adsorbent is an important parameter in the adsorption process applications. The study was evaluated using 100 mg of the α-HNPs that resulted after the adsorption process. The regeneration step was proceeded using 200 mL of anhydrous ethanol at solution pH 2.0 for 12 h at 100 °C with constant stirring at 1500 rpm under the reflux system. Subsequently, it was filtered and dried in the oven at 150 °C overnight. The re-adsorption process was tested after generation to assess regeneration efficiency.

#### Azr-wastewater purification approach

The sample was collected from the pharmaceutical wastewater of production machine rinse from a pharmaceutical industrial factory in (Cairo, Egypt). Initially and before the adsorption process application, the physicochemical parameters as solution pH, conductivity, total dissolved solids (TDS), and HPLC assay were analyzed. For the adsorption test, in a 100 mL beaker; a 25 mL of real pharmaceutical wastewater samples that were previously filtered through a nylon membrane filter of 0.45 µm, the α-HNPs were added to get a concentration of 8.0 mg/mL with continuous stirring for 12 h at 500 rpm and temperature at 313 K. Consequently, the sample was filtered using nylon filter paper in 0.45 µm, then an additional filtration step was performed using syringe filter 0.2 µm before introduction to the HPLC instrumental system for analysis.

#### Zero-point charge (pH_zpc_) of Azr@α-HNPs

The zero-point charge is known as the pH solution where a neutrally charged nature of the surface of α-HNPs. The used pH drift method could be derived as previously reported by Al-Hakkani et al.^29^. The zero-point charge could be estimated using the intersection of the curve between the initial and final pH solution of the standard and test solution that contains Azr*@*α-HNPs*.*

The fraction conversion relationship between the pH value and its mV value could be calculated as the following equations:

18 $$Fraction\,pH \, = \, 7.00 \, - \, \left( {estimated\,pHzpc} \right),$$

19 $$mV = \, Fraction\,pH \, \times \, 57.14.$$

#### Antibacterial activity

The antibacterial activity of the Azr*@*α-HNPs was conducted against four bacterial strains; *Bacillus* *subtilis* (*B*. *subtilis*) & *Staphylococcus* *aureus* (*S.* *aureus*) as Gram-positive bacteria and *Escherichia* *coli* (*E.* *coli*) & *Pseudomonas* *aeruginosa* (*P.* *aeruginosa*) as Gram-negative bacteria. The bacterial species were obtained from the Microbiology lab, Faculty of Science, New Valley University, El-kharga, Egypt. The test activity was conducted as the previously reported approaches^7,29^ using Azr (200 µg/mL) as a positive control, while Azr*@*α-HNPs were added into the wells at two different concentrations (50 and 200 µg/mL). The incubation at 30 °C for 24 h for plates was conducted. The inhibition zones were investigated in mm and recorded.

#### Anticancer activity

MCF7, HepG2, and HCT116 were supplied by Science way company that purchased from the *ATCC* and Asterand. MTT assay test was conducted to evaluate the cell proliferation as reported in the previous literature via Al-Hakkani et al.^7,29^. Cytotoxicity and viability profiles using the as-bioengineered Azr*@*α-HNPs were conducted in the range of concentration (31.25–1000 μg/mL) against three different cell lines (MCF7, HepG2, and HCT116). The viability (%) and cytotoxicity (%) could be calculated using the following equations:

20 $$Viability \, \left( \% \right) \, = \, 100 \, {-} \, \left[ {\left( {Control\,O.D \, {-} \, Test\,O.D} \right)/ \, Control\,O.D \, \times \, 100} \right],$$

21 $$Cytotoxicity \, \left( \% \right) \, = \, 100 \, - \, Viability \, \left( \% \right).$$

#### Antiviral activity

##### Mode of action against SARS-CoV-2 “Mechanism of virus inhibition via Azr*@*α-HNPs”

Possible mechanism of hCoV-19/Egypt/NRC-03/2020 (Accession Number on GSAID: EPI_ISL_430820) virus inhibition by the Azr*@*α-HNPs was studied at three different levels as follows:

1. Viral replication

The assay was implemented in a 6 well plate where Vero E6 cells were cultivated as (10^5^ cells/mL) for 24 h at 37 °C. Virus was diluted to give 10^3^ PFU/well and applied directly to the cells and incubated at 37 °C for 1 h. Unabsorbed viral particles were removed by washing cells three successive times using supplements free-medium. Azr*@*α-HNPs were applied at different concentrations in the range (31.2–250 µg/mL). After 1 h contact time, 3 mL of DMEM medium supplemented with 2.0% agarose was added to the cell monolayer. Plates were left to solidify and incubated at 37 °C till the appearance of viral plaques. Cell monolayers were fixed in 10.0% formalin solution for 2 h, and they were stained with crystal violet. Control wells were included additionally Vero E6 cells were incubated with the virus and finally, plaques were counted. The reduction percentage in plaques formation and the viability percentage in comparison to control wells could be calculated as the following:

22 $${\text{Inhibition }}\left( \% \right) \, = \, \left( {{\text{Plaques}}\,{\text{in}}\,{\text{viral}}\,{\text{control }} - {\text{ Plaques}}\,{\text{in}}\,{\text{teste}}} \right)/{\text{Plaques}}\,{\text{in}}\,{\text{viral}}\,{\text{control }} \times { 1}00,$$

23 $${\text{Cytotoxicity }}\left( \% \right) \, = \, \left( {{\text{Control}}\,{\text{cells}}\,{\text{absorbance }}{-}{\text{ Test}}\,{\text{cells}}\,{\text{absorbance}}} \right)/{\text{Control}}\,{\text{cells}}\,{\text{absorbance }} \times { 1}00,$$

24 $${\text{Viability }}\left( \% \right) \, = { 1}00 \, - {\text{Cytotoxicity }}\left( \% \right).$$

2. Viral Adsorption

VeroE6 cells were cultivated in a 6 well plate (10^5^ cells/mL) for 24 h at 37 °C. Azr*@*α-HNPs were applied at different concentrations in the range (31.2–250 µg/mL) of 200 µL medium without supplements and co-incubated with the cells at 4 °C for 2 h. Unabsorbed Azr*@*α-HNPs were removed by washing cells three successive times with supplements free-medium. Then virus was diluted to give 10^3^ PFU/well and co-incubated with the pretreated cells for 1 h followed by adding 3 mL DMEM supplemented with 2% agarose. Plates were left to solidify and then incubated at 37 °C to allow the formation of the viral plaques, fixed and stained as mentioned in viral replication mode. The reduction percentage in plaques formation was calculated as Eq. (22) in comparison to control wells where untreated cells were directly infected with the virus.

3. Virucidal

The assay was conducted in a 6-well plate where VeroE6 cells were cultivated (10^5^ cells/mL) for 24 h at 37 °C. A volume of 200 µL serum-free DMEM containing 10^3^ PFU/well. The virus was added to the concentrations of Azr*@*α-HNPs in the range (of 31.2–250 µg/mL). After 1 h incubation, the mixture was diluted using serum-free medium 3 times each tenfold still allowing the existence of viral particles to grow on cells. Then a volume of 100 µL of each dilution was added to the cell monolayer. After 1 h contact time, DMEM overlayer was added to cell monolayer. Plates were left to solidify and then incubated at 37 °C to allow the formation of viral plaques, fixed and stained as previously mentioned. The percentage of the reduction in plaques formation in comparison to control wells was calculated according to Eq. (22).

## Results and dissection

### Interactive effect of solution pH change on the adsorption process

The pH of the adsorbate solution has a strong impact on the adsorption process which directly affects the adsorbate solubility and dissociation facilitating the interactive relationship with the adsorbent^23^. Azr pKa equals about 8.5; this means, at a pH of 8.5 of Azr; 50% is ionized and 50% is unionized^43^. At lower pH solutions 2.0–4.0 the adsorption removal process was low which started from 20.8% and slightly increase, reaching 22.2% of Azr removal within adsorption capacity alteration from 20.8 to 22.2 mg/g. This means that at this pH range the removal efficiency is not pH-dependent which is agreed with previously reported work by Siavash et al*.*^26^ which may be attributed to electrostatic repulsion force between the Azr and α-HNPs surface. An observable increment in the removal % of the Azr with about 13% at solution pH 6.0 corresponded to an adsorption capacity of 34.6 mg/g. Moreover, the electrostatic attraction force between the Azr drug and the α-HNPs surface was progressively raised to the highest level of pH adsorption greater than 8.0. This behavior was realized in the current study, especially at pH 10.0 where the removal % near about 79% with an adsorption capacity of 78.8 mg/g.

Also, the Azr increasing trend from pH 6.0–10.0 may be assigned to the pH (zero-point charge) of the α-HNPs which is lower than the pKa of Azr. pH (zero-point charge) of α-HNPs was found to be 5.2 as previously determined^29^ so, at lower pH of 5.2; the surface of α-HNPs will be protonated and become positively charged in nature. On the other hand, at pH higher than 5.2; deprotonation of the surface of α-HNPs became negatively charged so, more electrostatic attraction force will be obtained between Azr molecules and the α-HNPs surface^26^.

Another assumption may be considered for the interpretation of the adsorption process between α-HNPs and Azr that results from the molecular structure of the Azr as shown in Fig. 1. There is anybody cannot deny the probable interaction that could be occurred through π–π interaction or π–π stacking (like benzene rings or carbonyl groups) between the functional groups that could be present on the adsorbent surface during the biosynthesis of the α-HNPs and the Azr^44^. Also, hydrogen bonding may be formed especially since the Azr contains many hydroxyl groups.

### Adsorbent dose effect

The adsorbent mass effect manifested a significant influence against the removal of Azr by α-HNPs that displayed an improved adsorption potent of the Azr by raising the dosage of adsorbent α-HNPs to 150 mg. This increase may be due to the higher surface areas of the α-HNPs 28.01 g/m^2^ which generated a large number of available active sites for the adsorption system^29^.

The adhesion trapping locations on the adsorbent surface were also limited and not large enough to realize the high adsorption of the Azr when the adsorbent mass was low, so the adsorption performance was underprivileged. The rise in the total mass dose of α-HNPs contributes to an improvement in the increase of the active sites for the Azr adhesion which indicated that more of the Azr was adsorbed on the surfaces of α-HNPs. Any mass increase beyond 150 mg of the adsorbent was followed by a small increase in the removal capacity as revealed, where the removal % trend was 78.8% → 86.1% → 90.5% → 91.6% of the adsorbent mass effect 50 mg → 100 mg → 150 mg → 200 mg respectively.

### Isothermal models study and adsorbate concentration effect

**Figure 2:**
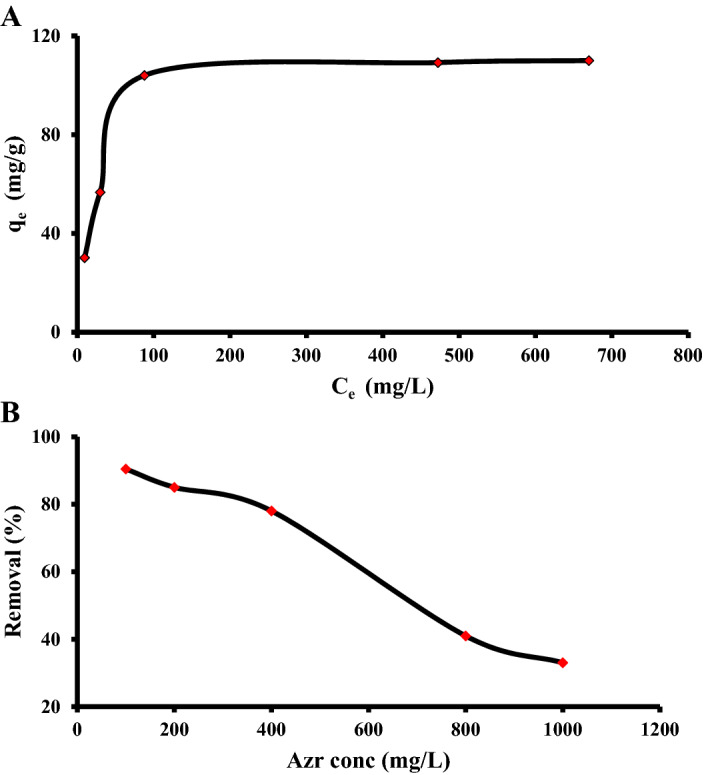
(**A**) Azithromycin adsorption capacity against its equilibrium concentration. (**B**) Azithromycin adsorption removal (%) against its initial concentration.

Figure 2A revealed an improvement in the adsorption potential of α-HNPs for the Azr at different concentrations. The adsorption capacity plateau manifested an observable change increase from 30.2 to 104.0 mg/g at the concentration of Azr from 100 to 400 mg/L. Subsequently, relative stability with a minimum slight increase in the adsorption capacity was demonstrated from 104.0 to 110.0 mg/g against Azr*'s* initial concentration of 400–1000 mg/L.

The unexpected drop in the percentage of Azr removal occurred after the third point 400 mg/L concentration Fig. 2B where the removal percent behavior, decreased to 90.46% → 85.03% → 78.03% → 40.96% → 33.01%. This means the most convenient concentration of the adsorbate that can be used was 400 mg/L.

The adsorption data were analyzed as in Table 1 revealed using different isotherm models to assess and determine the most fitted isothermal model that could describe the relationship between the adsorbent/adsorbate in the current adsorption study.

**Table 1:** Investigated isothermal data according to the different applied models.

Item	Isothermal models			
	Langmuir	Freundlich	Temkin	D–R
R^2^	0.9992	0.8278	0.8645	0.8437
Model parameter	q_L_ = 114.05	n = 3.5	A_T_ = 0.814	q_m_ = 96.2
	k_L_ = 0.045	1/n = 0.288	B_T_ = 18.8	β = − 2.04 × 10^–5^
	R_L_ = 0.027	k_F_ = 19.8	b_T_ = 129.3	E_D_ = 156.5

**Figure 3:**
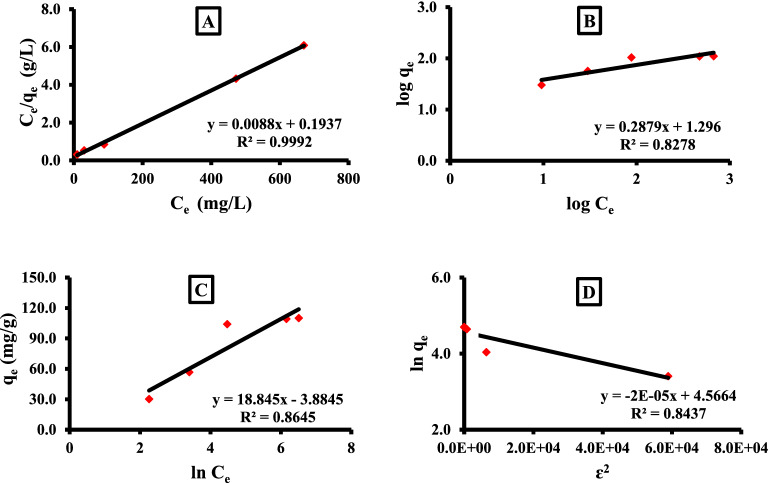
Isothermal models: (**A**) Langmuir, (**B**) Freundlich, (**C**) Temkin, (**D**) D–R.

Langmuir isotherm model was the most convenient to express the adsorption process, where the “R^2^” was found to be closed for the unit (0.9992) Fig. 3. Also, the calculated Langmuir monolayer q_L_ (114.05 mg/g) on the homogenous α-HNPs surface was found to be around the experimental q_e_ (104.04 mg/g). So, the adsorption type was classified as a favorable in behavior, where the R_L_ equaled 0.027 which was more than zero and less than the unit. As the results were be manifested in Table 1.

The use of the different isotherm models not only to find and describe the best isotherm assumption, but several significant parameters can be derived to help in understanding more details about the adsorption process of nature.

The Temkin isotherm model also was investigated to analyze the collected experimental data. According to Temkin's assumption “the heat of the adsorption will decrease by rising the adsorbent mass”. The parameter b_T_ is a constant that is related to the heating of the adsorption process. According to the Temkin model, if the b_T_ value is > 80 kJ/mol that means the adsorption is chemisorption in nature^23,28,45^; in our investigations, the b_T_ was found to be 129.3.

As we can see using the Freundlich model, it can be used for assuring that the favored and desirable of the adsorption process through n parameter if it was located in between 1 and 10^23,28,45^; it was found to be equal to 3.5. Also, the 1/n value was found to be 0.288; which revealed that the adsorption was not close to zero. So, the adsorption did not follow the Freundlich model assumption as “heterogeneous for the surface energy of the binding active sites with reversible adsorption at multilayer formation”. The high k_F_ (19.8) value indicated that a high adsorption capacity occurred^46^.

The D–R isothermal model also was tested and provided us with the average adsorption energy for each Azr molecule adsorbed using α-HNPs “free energy (E_D_)”. It was found to be 156.5; since the higher value of E_D_ is greater than 80; it is indicating that the adsorption can be expressed as a chemisorption type^26^.

To determine the advantage of using of α-HNPs as a good adsorbent for Azr removal from contaminated wastewater, the maximum capacity of adsorption for α-HNPs and other adsorbents against Azr should be conducted. Table 2 summarizes the Azr adsorption maximum capacities for various adsorbents including α-HNPs that are dedicated to the present study. It was observed that the α-HNPs can be used as an effective removal adsorbent agent for the Azr-contaminated environment wastewater. Green-fabricated α-HNPs revealed a moderated maximum adsorption capacity for Azr compared with some of the reported adsorbents, indicating that it can be used as a promising adsorbent agent for the Azr removal from the contaminated aquatic environments, especially as wastewater of hospitals and pharmaceutical industries.

**Table 2:** Azr adsorption maximum capacities for various adsorbents.

Adsorbent	Maximum capacity (mg/g)	References
Zeolite analcime	407.54	^24^
MCM-41 by microwave	208.54	^25^
MCM-41 by hydrothermal	235.58	
ZnO NPs	160.4	^28^
α-HNPs	**114.05**	**Current study**
Raw nano diatomite	68.0	^26^
Modified nano diatomite	91.7	
GO@Fe_3_O_4_/ZnO/SnO_2_	9.375	^20^
PAC/Fe/Si/Zn nanocomposite	7.93	^47^
Nanosized faujasite zeolites crystals-1	8.5	^27^
Nanosized faujasite zeolites crystals-2	7.0	

Significant value is in bold.

### Kinetic studies

The kinetic profile of Azr adsorption via the α-HNPs at different time intervals was investigated. The results manifested that the adsorption of Azr onto the α-HNPs surface was directly time-dependent. It was revealed that most of the Azr adsorption occurred within a time of 60 min. After the first hour, slight increases in the Azr uptake, and approximately the adsorption process were relative-stable practically.

The adsorption process was increased rapidly in the first 10 min due to the presence of many available free active binding sites on the α-HNPs surface and the high adsorbate concentration in the solution. After this time, little unbounded active sites can be available at the adsorbent surface. At the time in the range, of 60–300 min, a slowly increased rate in the Azr removal was observed.

**Figure 4:**
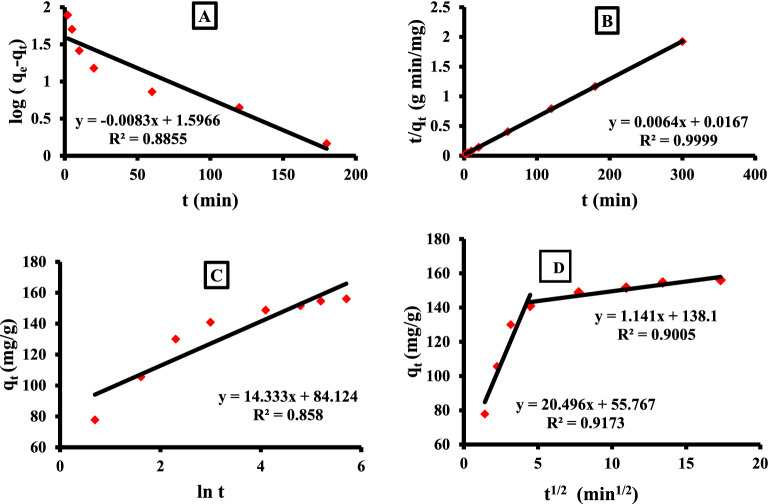
Kinetic models: (**A**) Lagergren, (**B**) McKay-Ho, (**C**) Elovich, (**D**) Weber and Moris.

Table 3 summarizes the obtained results of the applied different four kinetic models. The obtained data revealed that; the pseudo-second-order kinetic model was the most fitted as the R^2^ was found to be the closest to the unit, Fig. 4 and Table 3. The four kinetic models could be arranged in order according to less of the correlation coefficient of the square of the Pearson product-moment “R^2^” as the following; pseudo-second-order > intraparticle diffusion > pseudo-first-order > Elovich. Also, the calculation of the maximum adsorption capacity q_e_ value was found to be 157.0 mg/g which was very close to the experimental value of 156.1 mg/g at 300 min for the adsorption reaction time.

**Table 3:** Different parameters of the applied kinetic models.

Item	Kinetic models			
	Lagergren pseudo-first-order	McKay and Ho pseudo-second-order	Weber’s and Moris’s intraparticle diffusion	Elovich
R^2^	0.8855	0.9999	R^2^_1_ = 0.9173	0.8580
Model parameter	K_1_ = 0.019	K_2_ = 0.002	K_int(1)_ = 20.5	*α* = 5073.1
	Calculated q_e_ = 39.5	Calculated q_e_ = 157.0	C_1_ = 55.77	β = 0.07
	Experimental q_e_ = 156.1		R^2^_2_ = 0.9005	
			K_int(2)_ = 1.14	
			C_2_ = 138.1

In the pseudo-second-order and Elovich models, both of them supposed that “the chemical adsorption is a result of the prevailing adsorption process”^27^, and the adsorption efficiency is proportional to the number of free active sites subsequently, occupied by the adsorbent. This was found to be compatible with the obtained results from the isothermal study, which interpreted that the adsorption process was chemisorption in nature. Also, the same behavior of the adsorption was found to be compatible with our previous approach while the Cfx was adsorbed at the surfaces of the α-HNPs as a chemisorption type^48^. On the other hand, the high affinity of Azr against α-HNPs may be attributed to complex formation. It was reported that the Azr can act as a bidentate ligand to form a complex with various metal ions including Fe (III) ion species especially at high temperatures and [Fe(Azr)_2_(H_2_O)_2_]2Cl_2_·2H_2_O complex was formed^49^.

### Thermodynamic study

The Azr uptake thermodynamic study by α-HNPs was proceeded at four different temperature degrees in the range 293–323 K to recognize the nature of the adsorption process's robustness and its practicability.

At the maximum temperature was the highest percentage of Azr removal, which had adopted an endothermic direction in the adsorption process. Rising the temperature beyond 313 K has corresponded to relative stability in the removal percentage of the Azr showing that the maximum critical temperature can be expressed at 313 K. This finding was ensured by experimental thermodynamics, as seen in Table 4.

**Table 4:** Thermodynamic parameters of the Azr adsorption using α-HNPs.

Temperature (K)	Removal (%)	ΔG (kJ/mol)	
293	78.0	− 3.1	∆H = + 87.6 kJ/mol ∆S = + 307.6 J/mol K
303	84.6	− 4.3	
313	97.8	− 9.8	
323	98.6	− 11.5	

Table 4 revealed a direct proportionality between the increase in temperature and the removal rate of the adsorption. This behavior may be attributed to the chemical interaction between the activated sites of the α-HNPs surface or the capping layer onto the α-HNPs surface that were adsorbed at the nano-phase biofabrication.

The negative sign of the free energy change ΔG confirmed that the adsorption type could be spontaneously^50^. Also, the positive signs of ΔH and ΔS assured that the adsorption behavior was obeyed by the endothermic class^51^. This clarified the cause of the increase in the removal rate against the increase in the temperature. The enthalpy function ΔH was calculated and it was found to be 87.6 kJ/mol which confirmed the chemisorption nature where ΔH ≥ 80 kJ/mol^23,28,45^ which was found to be agreed with the previously obtained results from the isothermal and kinetic studies.

### Regeneration process capability of the adsorbent

**Figure 5:**
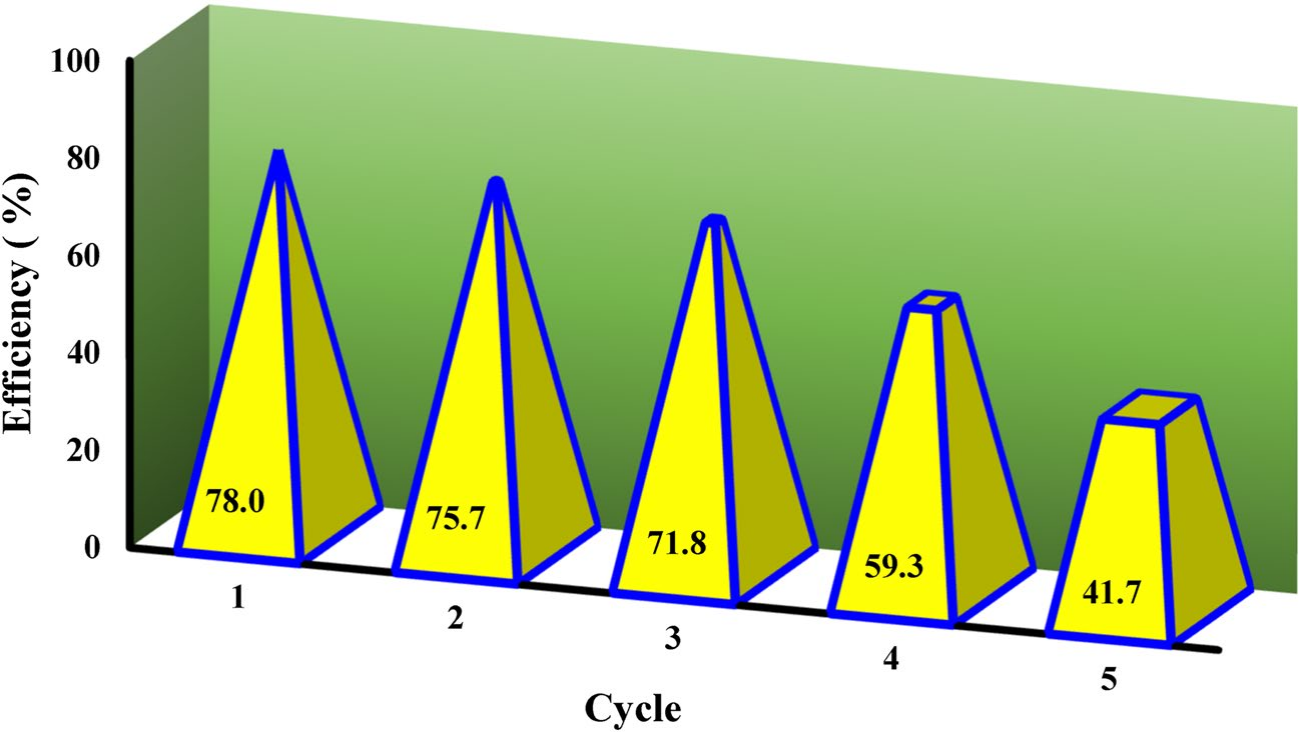
Regeneration trend of the *α-HNPs.*

The reusability process capability of the α-HNPs adsorbent was studied for 5 cycles (Fig. 5). Up to the third cycle; the regenerated adsorbent was found to be efficient. Subsequently, the performance decreased sharply and reached approximately 59.3%. The result may be attributed to the reduction of the available active binding sites on the adsorbent surface during cycles of regeneration especially, the adsorption was chemisorption type. So, the α-HNPs may be reused a maximum of three times with a reasonable performance of Azr removal according to the mentioned dedicated procedures.

### Azr-wastewater purification approach

The physicochemical characteristics of wastewater and assay of the Azr before and after adsorption treatment were evaluated and listed in Table 5. The operating procedures manifested, that the efficiency of the adsorption method in the industrial, and pharmaceutical wastewater treatment using α-HNPs as a promising adsorbent.

**Table 5:** Wastewater physicochemical characteristics and assay of the Azr before and after treatment.

Characteristic parameter	Before	After
Concentration (mg/L)	156.3	Not detected
Conductivity (μS/cm)	415.5	205.1
TDS (mg/L)	221.8	105.7
pH	10.76	8.33

### Zero-point charge (pH_zpc_) of Azr@α-HNPs

An increase in the final solution pH in the test solution of Azr*@*α-HNPs suspension was the following “2.0 → 2.3, 4.0 → 4.4, 6.0 → 6.7”. While the final pH value of the test solution from pH 8.0 to 12.0 was found to be decreased to be less basic; that might be attributed to the neutralization between the alkaline adsorbed Azr species at the acidic surface of α-HNPs. The pH curve of the Azr*@*α-HNPs crossed the straight line at the pH value equaled pH_zpc_ 7.8. The surface charge of Azr*@*α-HNPs suspension could be determined using Eqs. (18) and (19) which were found to equal to − 45.71 mV.

Al-Hakkani et al*.*^7,52^ reported that the suspended particles had good stability if their charged surface passed the critical value ± 25 mV. The high negativity of the charged surface may be attributed to the polyphenolic constituents that act as stabilizing/capping agents. Highly positive/negative values onto the charged surface generate major repulsion forces, whilst repulsion between particles with the same electrical charge inhibits the particle agglomeration and hence it gives good dispersibility.

### FT-IR analysis

**Figure 6:**
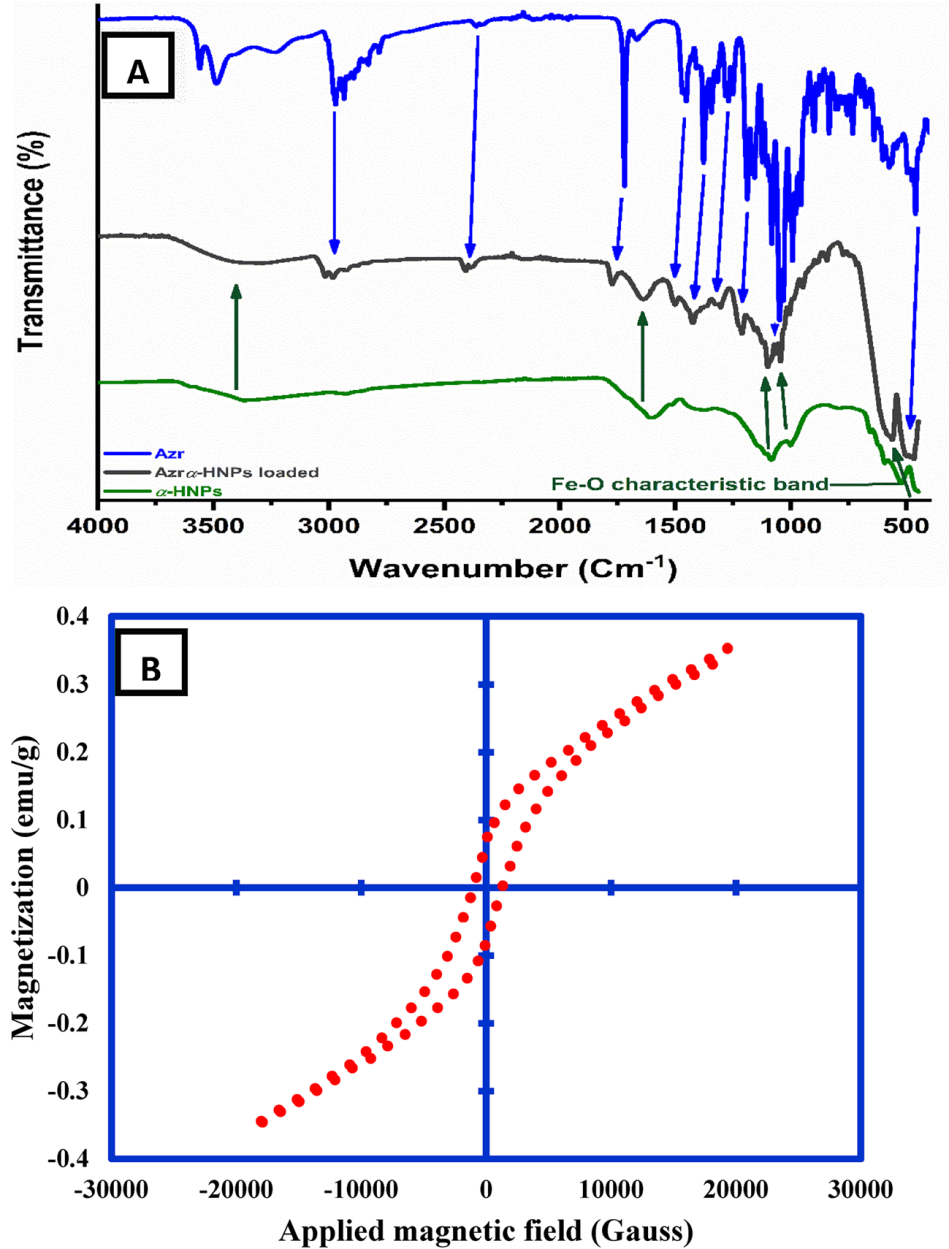
(**A)** FT-IR spectra: *Azr*, α*-HNPs*, *Azr@α-HNPs*, (**B**) The magnetization curve (VSM) of the as-prepared *Azr@α-HNPs*.

FT-IR analysis plays an important role to confirm the adsorption and participation of some of the functional groups that are adsorbed at the surface of the α-HNPs to form Azr*@*α-HNPs Fig. 6A ^7,14^. As we can see there are many functional groups dedicated to Azr that have clear contributions from the adsorption process onto the surface of α-HNPs*.* This was manifested especially in the fingerprint region of Azr (1600–500 cm^−1^; blue arrows) except in the Fe–O band (522 cm^−1^)^7,29^. These constituents gift the possibility to further interaction between the α-HNPs and Azr molecules through double bonds, a hydrogen bond, or electrostatic interaction.

### Magnetic measurements

The VSM of the as-biosynthesized Azr*@*α-HNPs was measured and the saturation magnetization (M_s_) was determined as 0.353 emu/g as shown in Fig. 6B, while the M_s_ of the α-HNPs alone was found to be 0.445 emu/g^29^. It was believed that the encapsulation of α-HNPs via the adsorbed Azr molecules may be the cause of decreasing in the M_s_ value. The deterioration of the magnetization energy may be a result of the formation of the adsorbed capping layer at the surface leads to Ref.^29^. This assumption also was confirmed by Ansari et al*.*^53^ who reported in their work the value of the M_s_ that was 0.67 emu/g for the biosynthesized porous α-Fe_2_O_3_ NPs using *Nepeta* *cataria* L. leaves extract.

### TEM analysis

**Figure 7:**
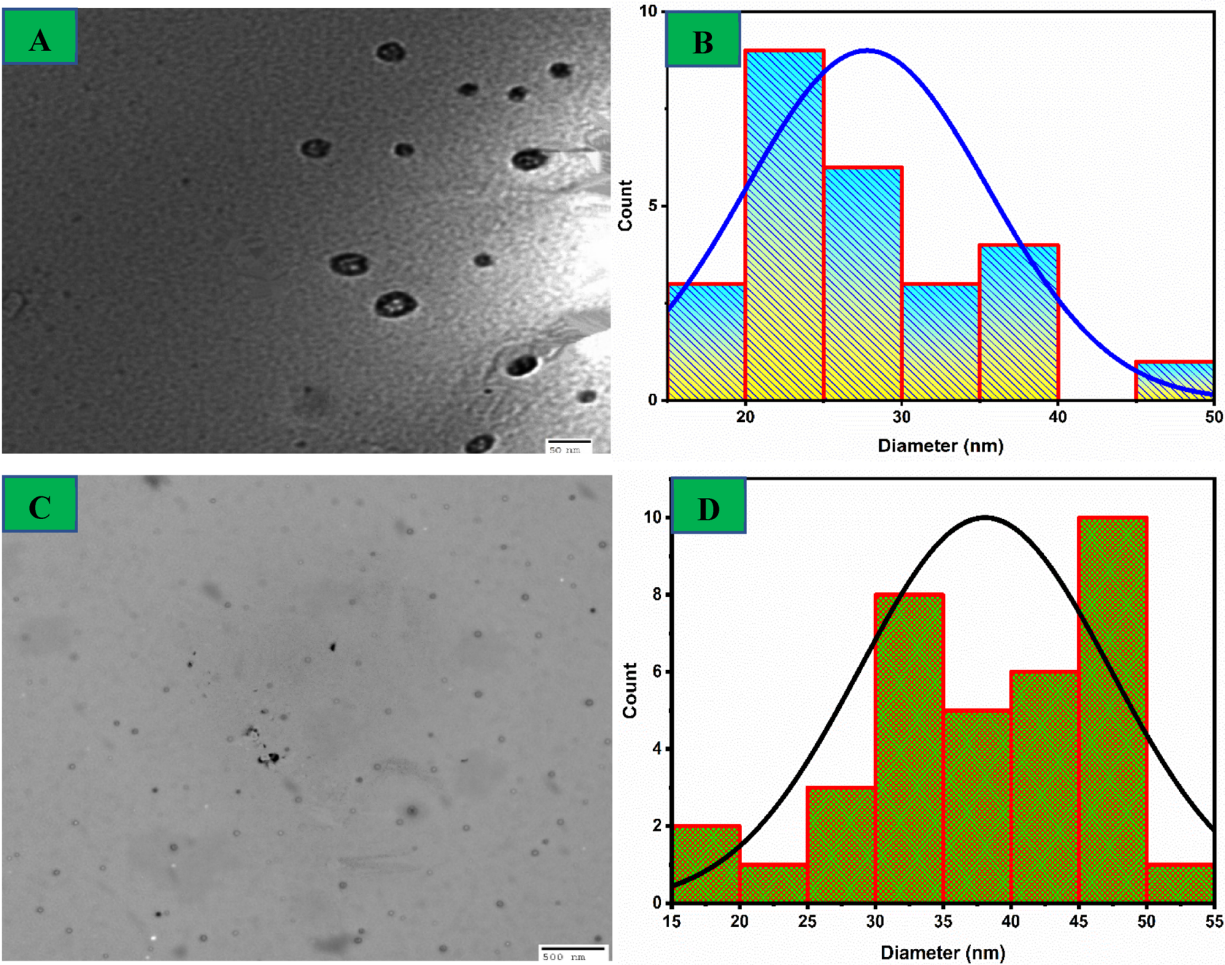
TEM images and particles distribution of *α-HNPs* (**A**,**B**), and *Azr@α-HNPs* (**C**,**D**).

The α-HNPs and Azr*@*α-HNPs TEM images were investigated as revealed in Fig. 7. Semi-spherical, spherical, and cubic monodispersed particles were conducted, and no considerable agglomeration was found. The average particle sizes of α-HNPs and Azr*@-HNPs* were recorded as 27.8 ± 7.7 nm and 38.1 ± 9.3 nm respectively, with a median of 25.9 nm and 39.2 nm at the lowest particle size of 17.7 nm and 16.4 nm, and maximum particle size of 49.0 nm and 50.5 nm for α-HNPs and Azr*@-HNPs* respectively. The encapsulating attraction force between both the Azr and α-HNPs surfaces might be the reason for the increase in the particle sizes of α-HNPs via the Azr adsorption technique^7^.

### Surface morphologies

**Figure 8:**
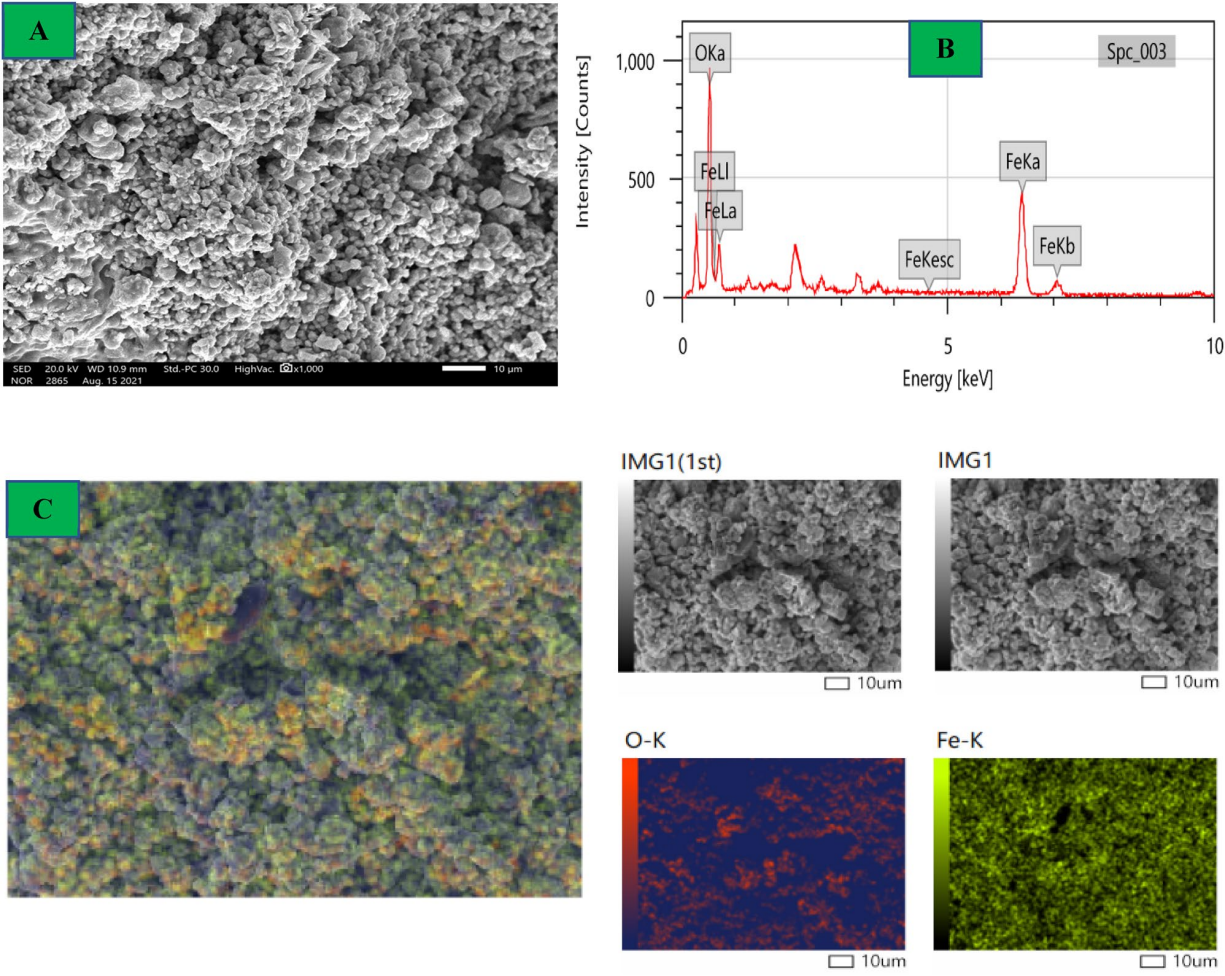
*α-HNPs* morphological analysis (**A**) SEM, (**B**) EDX, (**C**) Mapping.

**Figure 9:**
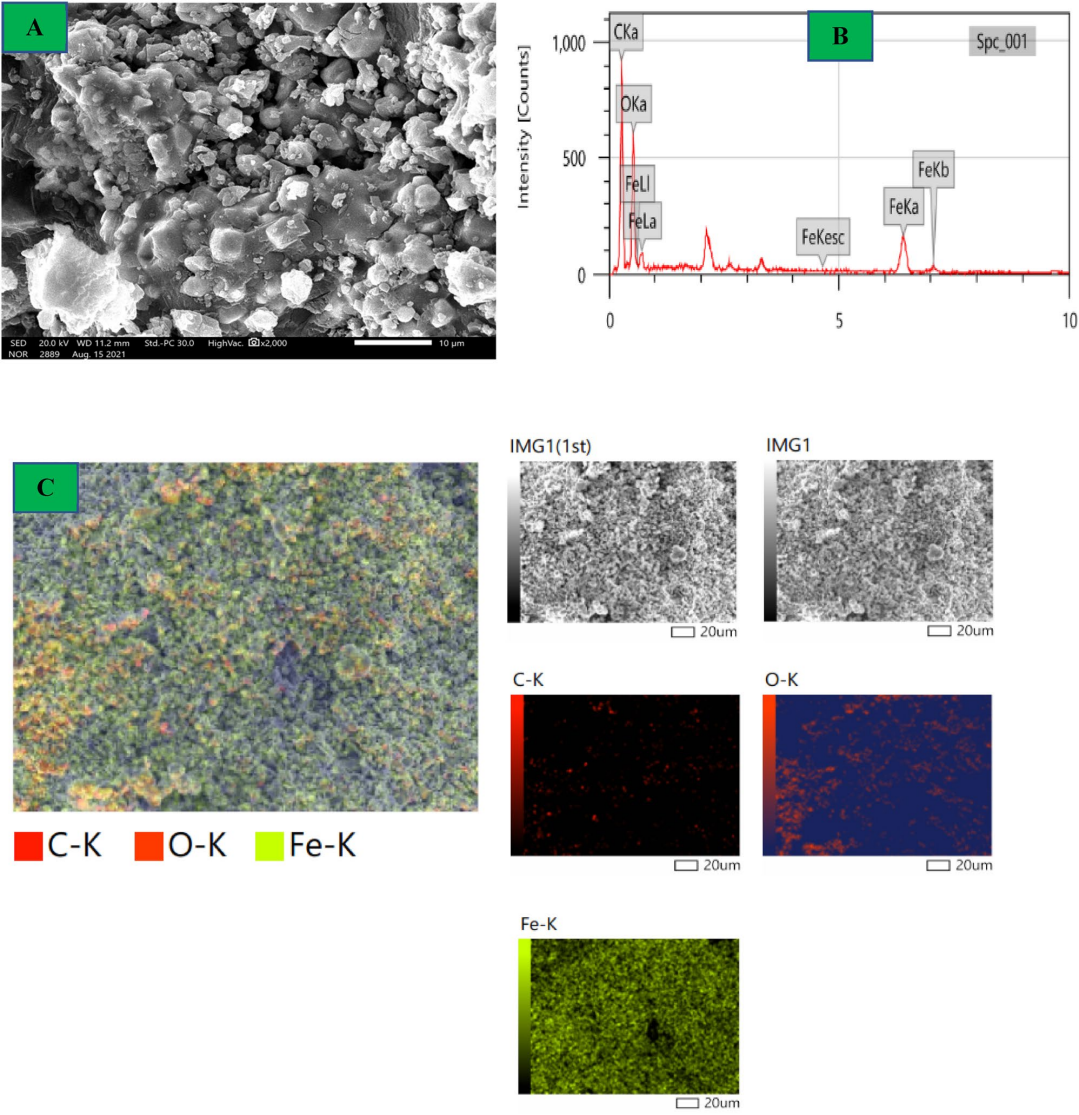
*Azr@α-HNPs* morphological analysis (**A**) SEM, (**B**) EDX, (**C**) Mapping.

Indeed, most properties and the use of NPs are based on their shape and size. It’s very clear the surface morphologies variations After Azr adsorption onto the α-HNPs surfaces (Figs. 8, 9). The adsorbed Azr molecules are the main cause of the changes between of α-HNPs and Azr*@*α-HNPs surfaces. SEM, EDX analyses, and mapping morphologies revealed and assured the adsorption process of Azr as Fig. 9C that manifested the most element distribution as carbon that represents the principal element of the Azr molecules. The clear homogeneity of carbon atoms indicated that the α-HNPs surface homogeneity that confirms the adsorption process follows Langmuir isothermal model. The encapsulated α-HNPs *via* Azr could be confirmed their further biological activity role, especially in antiviral, anticancer, and antibacterial applications.

### Antibacterial activity

The inhibition zone values in mm were manifested in Table 6.It's clear for a man that the anti-bacterial activity is Azr*@*α-HNPs concentration-depended as variation in the inhibition zone values. The most susceptible microbial species against Azr*@*α-HNPs at (200 µg/mL) were found to be 38, 32, 46, and 43 mm for *E.* *coli*, *P.* *aeruginosa*, *B.* *subtilis*, and *S.* *aureus* respectively. The adsorbed Azr on the α-HNPs revealed a synergistic effect in the case of Gram-positive bacteria over Gram-negative compared with α-HNPs alone as in the current study and the previously reported approaches^7,29^. These results also were found to be agreed with the conducted study via Morteza et al.^54^ using Azr NPs as an antibacterial agent against several types of bacteria. They reported that the prevalence of a double membrane might prohibit some structures from cell penetration, partly because of the reasons why Gram-negative bacteria are typically more antibiotically resistant than other Gram-positive bacteria.

**Table 6:** Antibacterial activity of the Azr*@*α-HNPs against different bacterial species.

Item	Inhibition zone (mm)							
	*E.* *coli*	*P.* *aeruginosa*	*B.* *subtilis*	*S.* *aureus*				
Azr (200 µg/mL)	26	18	21	15				

Item	α-HNPs	Azr*@*α-HNPs	α-HNPs	Azr*@*α-HNPs	α-HNPs	Azr*@*α-HNPs	α-HNPs	Azr*@*α-HNPs
200 (µg/mL)	24	38	20	32	22	46	19	43
50 (µg/mL)	13	17	9	12	10	16	10	19

In our previous study of the adsorbed Cfx at the α-HNPs surfaces at a concentration of 200 (µg/mL), Cfx@α-HNPs against the same bacterial species manifested a lower antibacterial activity in the case of *B.* *subtilis* *and* *S.* *aureus*. On the contrary, Azr*@*α-HNPs antibacterial activity against *E.* *coli* *and* *P.* *aeruginosa* showed lower activity the revealed by Cfx@α-HNPs^48^.

Our approach showed excellent and satisfactory results as an antibacterial agent compared with a recent study reported by Alangari et al*.*^55^ where they used a concentration of 100 μL of the prepared α-HNPs against *E.* *coli*, B. *subtilis*, P. *aeruginosa*, and *S.* *aureus*. Where our α-HNPs *and* Azr*@*α-HNPs system manifested high potent against the same bacterial pathogenic species. Also, a green synthesis approach of α-HNPs using *Hibiscus* *rosa* *Sinensis* flowers revealed a lower antibacterial efficacy against each of *S*. *aureus,* *E.* *coli,* *P.* *aeruginosa,* and *K.* *pneumonia*^56^*.* Compared with the antibacterial activity reported in a recent study of the biofabrication of the α-HNPs using *Achyranthes* *aspera* extract at different concentrations of α-HNPs (20–50 mg/mL), the current study outperformed the reported compared approach^57^.

### Anticancer activity

The cell viability (%) for each of the cell lines individually was conducted using serial dilution in different concentrations of the as-biofabricated Azr*@*α-HNPs at [31.25–1000 µg/mL]. The examination was reported using a visible spectrophotometric beam at 560 nm (Eq. 20).

Albukhaty et al*.*^58^ also confirmed in their investigation of the Dextran-Coated super paramagnetic nanoparticles the potential for cancer detection and treatment via the unique characteristics of iron oxide using the Annexin V-FITC Apoptosis for Apoptosis estimation. Also, Ibrahim et al*.*^59^ assured the role of the nano-complexation use approach and effective treatment in the Caov-3 cancer cell line.

**Figure 10:**
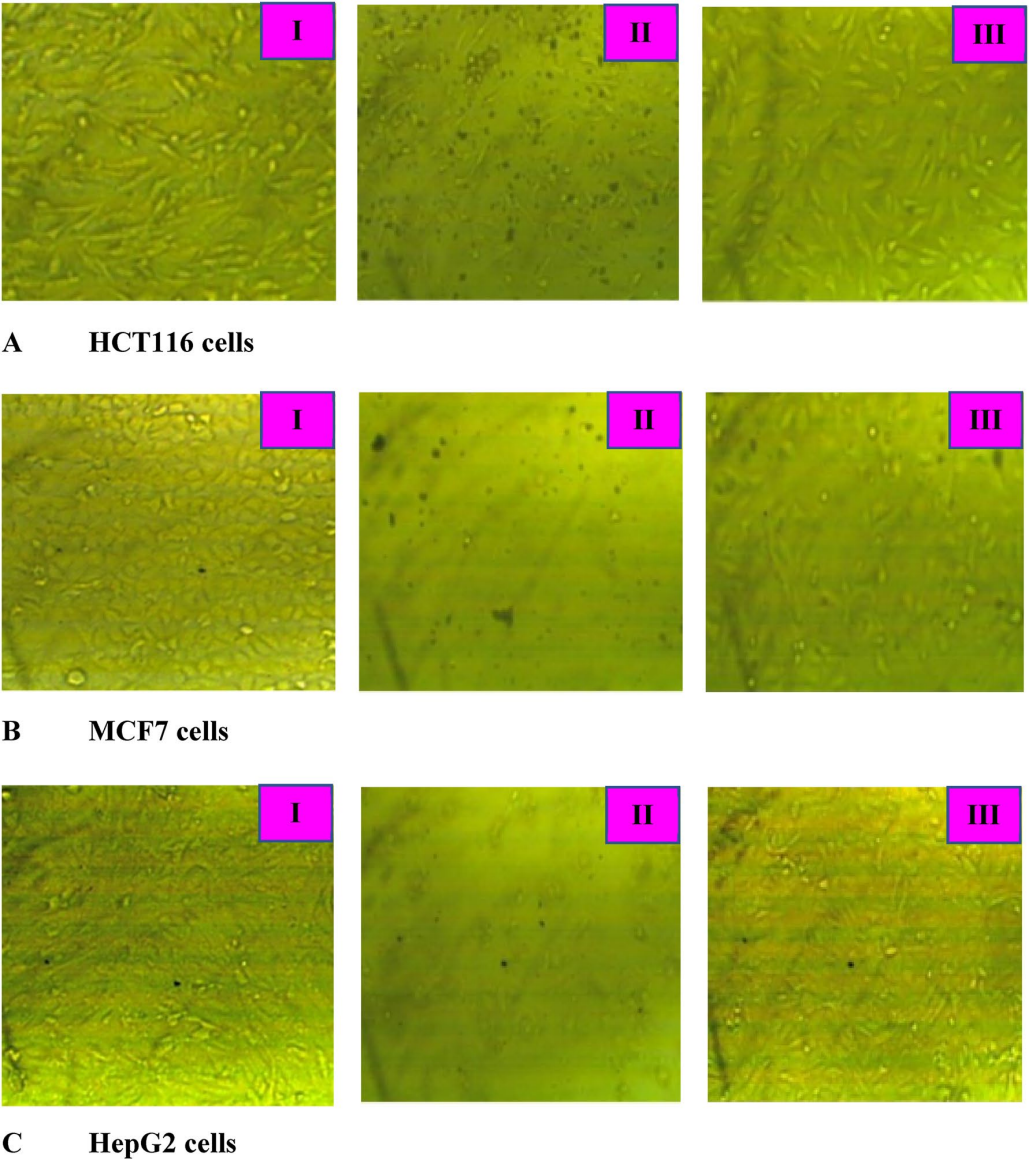
*Azr@α-HNPs* effect on different cell line at concentrations: (I) Zero μg/mL (II) 1000 μg/mL (III) 31.25 μg/mL.

The cytotoxicity effect of the Azr*@*α-HNPs against MCF7, HepG2, and HCT116 cell lines could be calculated according to Eq. (21). It was found to be extremely concentration-susceptible of Azr*@*α-HNPs also at the lower level of concentrations. Additionally, the cytotoxicity of cells was observed at 94.8%, 93.7%, and 95.0% at the concentration of 1000 µg/mL as MCF7, HCT116, and HepG2 cell lines respectively as shown in Fig. 10. IC_50_ was estimated for MCF7, HCT116, and HepG2 and was found to be 78.1, 93.4, and 81.7 µg/mL. The results manifested that the Azr*@*α-HNPs have admirable cytotoxic activity against different cell lines like the following sequence MCF7 > HepG2 > HCT116. These findings data is harmonized with the nanoparticles at 13.8 nm for the as-bioengineered Azr*@*α-HNPs, it could also be used as a highly-touted antitumor agent.

As we can observe, comparing the anticancer activity results of the Azr*@*α-HNPs against α-HNPs as it is; the adsorption of Azr onto the α-HNPs surface caused a reduction of the IC_50_ increasing the cytotoxicity effect of the α-HNPs against different cell lines as manifested in Table 7. This effect could be attributed to the anticancer role of Azr alone that was reported in previous works^6,60,61^. So, the present study confirms the synergistic Azr effect in the Azr*@*α-HNPs and its stellar efficacy as a promising anticancer agent.

**Table 7:** Anticancer activity IC_50_ [µg/mL] of α-HNPs, Cfx@α-HNPs, and Azr@α-HNPs.

Cell line type	α-HNPs	Cfx@α-HNPs	Azr*@*α-HNPs
MCF7	132.0	108.5	78.1
HepG2	189.0	117.7	81.7
HCT116	377.0	156.4	93.4
Reference	Al-Hakkani et al*.*^29^	Al-Hakkani et al*.*^48^	Current study

In a recent study that was conducted by Ansari et al*.*^53^, they reported that the green synthesized porous α-HNPs and the loaded nanosystem doxorubicin@HNPs had high potential as anticancer against melanoma cell lines via MTT. Also, they confirmed that the doxorubicin@HNPs had a better effect compared with porous α-HNPs alone.

Also, in a similar study using the hydrothermal approach as the green synthesis of α-HNPs*,* the anticancer activity against the HepG2, HeLa, and MCF7 cell lines were implemented showing the IC_50_ 71 μg/mL, 68.12 μg/mL, and 53.35 μg/mL respectively for the tested cell lines^55^. These findings confirmed the anticancer activities of the α-HNPs and their loaded nanosystem.

### Antiviral activity

The minimum inhibition concentration of the substance to inhibit 50% of the pathogen in-vitro (IC_50_) was used to assess the antiviral activity. IC_50_ values are depending on the measurement conditions as a type of inhibition, and ATP-enzymes concentrations as in competitive inhibition. On the other hand, CC_50_ is used to express the cytotoxic concentration of the substance causing 50% death of the viable cells in the host^62^. The therapeutic index (TI) is a value that indicates the drug substance selectivity (Azr*@*α-HNPs in our case). TI is used in assessing the utility of a drug substance, TI stresses the relevance of safety margins besides the efficacy, and it can be calculated as CC_50_/IC_50_.

TI should be ≥ 1 in the ideal situation where the IC_50_ value should be less than the CC_50_ value to assure that killing the pathogen before the host cells were affected, damaged, or killed via drug substance^63^. The higher values of TI are the safer for the drug and preferred the lower values. If the TI < 1 denotes the drug substance is inactive and the drug should have been properly dosed and any symptoms of drug toxicity closely monitored by the individual receiving the treatment.

**Figure 11:**
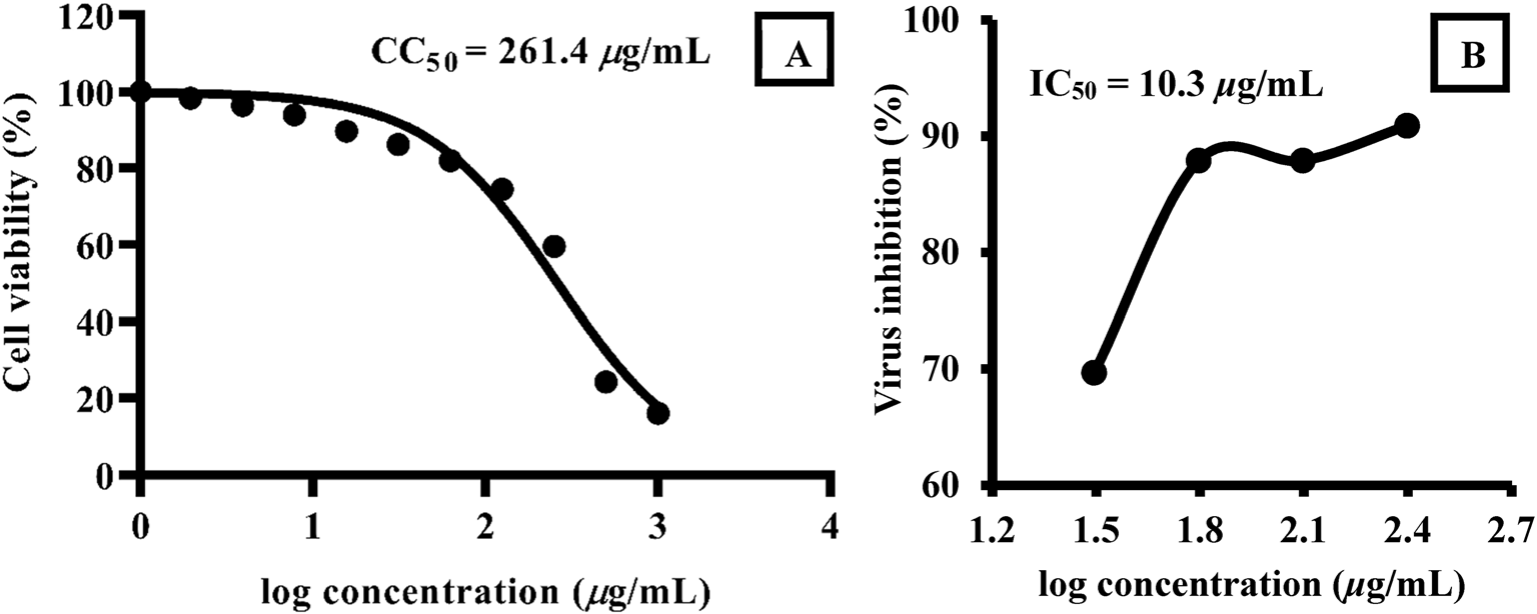
*Azr@α-HNPs* of: (**A**) Cell viability (%), (**B**) Virus inhibition (%) against SARS-Cov-2.

According to replication action mode results that revealed the Azr*@*α-HNPs have IC_50_ = 10.3 μg/mL and CC_50_ = 261.4 μg/mL Fig. 11. The inhibition curve showed a direct concentration-dependence of Azr*@*α-HNPs where the lower in concentration the lesser the antiviral activity. The results manifested that Azr*@*α-HNPs have high activity against SARS-CoV-2 in-vitro with a therapeutic index TI = 25.4.

The Azr*@*α-HNPs TI in the current study was found to be higher than that was reported in our previous study wherein the case of the Cfx*@*α-HNPs (TI = 2.6)^48^. The higher efficacy of the Azr*@*α-HNPs against SARS-CoV-2 comparing that was realized in the case of Cfx*@*α-HNPs may be attributed to the combination of the hyperactivity of the adsorbed Azr *at* the surfaces of the α-HNPs.

Viral risk reduction is a key and fundamental human health aim. SARS-CoV-2 viruses are now identified as spherical morphologies and are 70–140 nm in diameter. Surfaces of SARS-CoV-2 are spiky^64^. So, according to variation of the diameters of SARS-CoV-2 and our Azr*@*α-HNPs system that is in the interest of our preparation in terms of nanoscale subservience. It is believed that this circumstance will facilitate the penetration of the RNA of the virus and inhibits its pathogenic action. SARS-CoV-2 is considered one of the fiercest viruses that threaten human life on the earth. Therefore, researchers in various fields have paid attention to try finding materials that have a direct effect on inhibiting this damn virus.

Several detection and treatment techniques of SARS-COV-2 could be used as Faizah et al*.*^65^ reported as clustered regularly interspaced short palindromic repeats^66^, Molecular diagnosis of COVID-19^67^, SARS-CoV-2 genome-based diagnostics^68^, Next-generation sequencing and SARS-CoV-2 detection^69^, COVID-19 diagnosis using viral proteins^70^, Lateral flow assay^71^, SARS-CoV-2 detection, diagnosis, and drug delivery systems-based nanotechnology^72^.

In a recent similar study of zinc oxide nano-spray effect against SARS-CoV-2, El-Megharbel et al*.* reported that the TI was 0.56 < 1.0^64^. So, they recommended the use of ZnO NPs a potent disinfectant against SARS-Cov-2. This could be attributed to the probable toxicity of the prepared ZnO NPs onto the cellular host. On the other hand, the calculated TI in the present study revealed the safety of Azr*@*α-HNPs against the cellular host and the cytotoxic effect against the SARS-Cov-2.

Also, as the use of the nanoparticles as an antiviral agent, Horacio et al*.*^73^ introduced their study for using hygiene product as mouthwash and nose rinse. The applied concentration of the silver NPs was (0.6 mg/mL) showing a high impact in decreasing the SARS-CoV-2 infection rate.

The current in-vitro study demonstrated that the infected Vero E6 cells with the SARS-CoV-2 virus, when exposed to a concentration of Azr@α-HNPs in the range (31.2–250 µg/mL), showed viral activity reduction. Mode of action was conducted to determine the most suitable inhibition mechanism of the SARS-CoV-2 virus via Azr*@*α-HNPs using three modes as revealed in Table 8. Adsorption mode was found to be the smallest action mode for virus inhibition where the determined viral inhibition percentage was found to be 33.3% only at Azr*@*α-HNPs concentration of 250 µg/mL. The working control in the adsorption mode was at virus count (1.5 × 10^5^ PFU/mL) compared with (1.0 × 10^5^ PFU/mL) viral titer post-treatment. On the right hand, virucidal action mode revealed a moderated inhibition with viral inhibition of 40.0% at working virus control count (0.5 × 10^5^ PFU/mL) against (0.3 × 10^5^ PFU/mL) via 250 µg/mL of Azr*@*α-HNPs*.*

**Table 8:** Mode of action of Azr*@*α-HNPs against SARS-CoV-2.

Mode of action	Azr*@*α-HNPs Conc. (μg/mL)	Virus control (PFU/mL) × 10^5^	Viral titer post-treatment (PFU/mL) × 10^5^	Viral inhibition (%)
Virucidal	250	0.5	0.3	40
	125		0.6	0
	62.5		0.6	0
	31.2		1.0	0
Replication	250	3.3	0.3	90.9
	125		0.4	87.9
	62.5		0.4	87.9
	31.2		1.0	69.7
Adsorption	250	1.5	1.0	33.3
	125		2.2	0
	62.5		2.5	0
	31.2		2.8	0

In replication action mode, the reduction activity was found to be concentration-dependence where at a high concentration of 250 µg/mL, the reduction reached 90.9% when compared to the control sample after 48 h of incubation. Also, at a low concentration, 69.7% viral inhibition was achieved at 31.2 µg/mL of Azr*@*α-HNPs*.* So, the viral replication action mode could be used to interpret the inhibition (%) of SARS-CoV-2 virus growth via Azr*@*α-HNPs. So, the replication mode was found to be the fittest mode and could be used as a promising inhibition material for COVID-19 treatment where the Azr*@*α-HNPs are mainly targeting the viral replication.

The FDA has validated nanoparticles of iron oxides as biocompatible materials for anemia^74^. Some studies have demonstrated the antiviral properties of iron oxide nanoparticles in-vitro as; Rotavirus^75^, Dengue virus^76^, Zika virus^77^, and influenza virus (H1N1)^78^.

Moreover, we believe that the Azr*@*α-HNPs may be used in COVID-19 treatment giving admirable results, especially the Azr itself has been used actually in COVID-19 in the treatment protocol as WHO recommended at the first pandemic of the emerging coronavirus. Also, recent studies reported Azr efficacy in this regard as an antiviral agent for a wide range of different virus species (influenza, rhinovirus, enterovirus, Ebola, and Zika)^3,4,76,79,80^. Recent studies confirmed the efficacy of different nanoparticles in the early treatment of COVID-19 as nitric oxide, iron oxides, copper, silver, and gold nanoparticles of nanocomposites^76,81–87^. Also, Yasmin Abo-zeid and her co-authors recommended the clinical trials against COVID-19 in their study that manifested the molecular docking approach of hematite (Fe_2_O_3_) and magnetite (Fe_3_O_4_)^88^. Also, Computational approaches to predict ligand-receptor binding and structure-based drug design for COVID-19 management. This recommendation is according to the great ability of iron oxide nanoparticles for the production of highly reactive oxygen species.

## Conclusion

This study aimed to use the green biosynthesized α-HNPs to remove the Azr drug from the contaminated pharmaceutical wastewater. The output data of the adsorption studies showed that; the most suitable isothermal model was the Langmuir at R^2^ 0.9992, with a maximum adsorption capacity of 114.05 mg/g. On the right hand, the kinetic investigation confirmed that the adsorption reaction followed the pseudo-second-order. The adsorption behavior was found to be endothermic spontaneous via chemisorption type. The results manifested the utility of the use of the nano bioadsorbent α-HNPs as a promising agent in the remediation of the Azr wastewater contaminated environment. Azr*@*α-HNPs revealed a diversity in the Eco-biomedical activities as antibacterial, anticancer, and antiviral. The antibacterial study manifested a high synergistic impact of the adsorbed Azr at the α-HNPs surface, especially in the case of Gram-positive. Also, the compatibility of Azr*@*α-HNPs revealed an admirable effect as an anticancer agent against MCF7, HepG2, and HCT116 cell lines causing a reduction in the IC_50_ compared with the use of α-HNPs alone against the same cell lines. In a study considered the first of its kind, Azr*@*α-HNPs manifested an antiviral effect against the pathogenic SARS-Cov-2 with a selectivity safety factor TI equal to 25.4 that qualifies it for use as an alternative biomedicine treatment.

## Data Availability

The data used to support the findings of this study are included in the article.

## Abbreviations

α-HNPs: Biofabricated alpha hematite nanoparticles
Azr: Azithromycin
Azr@α-HNPs: Adsorbed azithromycin at the surface of the biofabricated alpha hematite nanoparticles
Cfx: Cefixime
NPs: Nanoparticles
MTT: Colorimetric assay for measuring cell metabolic activity
Mcf-7: Human breast carcinoma cells
HepG2: Human liver hepatoma carcinoma cells
HCT116: Human colorectal carcinoma cells
IC_50_: Half maximal inhibitory concentration
CC_50_: Half-maximal cytotoxic concentration
FT-IR: Fourier-transform infrared spectroscopy
O.D: Optical density
HPLC: High performance liquid chromatography
SARS-CoV-2: Severe acute respiratory syndrome coronavirus 2
COVID-19: Coronavirus disease pandemic
pH_zpc_: Zero-point charge
*B*. *subtilis*: *Bacillus* *subtilis*
*S.**aureus*: *Staphylococcus* *aureus*
*E.**coli*: *Escherichia* *coli*
*P.**aeruginosa*: *Pseudomonas* *aeruginosa*
TI: Therapeutic index
M_s_: Saturation magnetization
TEM: Transmission electron microscopy
SEM: Scanning electron microscopy
EDX: Energy dispersive X-ray
FDA: The United States Food and Drug Administration
